# Association between socioeconomic status and fertility among adolescents aged 15 to 19: an analysis of the 2013/2014 Zambia Demographic Health Survey (ZDHS)

**DOI:** 10.1186/s12978-021-01230-8

**Published:** 2021-09-10

**Authors:** Margarate Nzala Munakampe, Isaac Fwemba, Joseph Mumba Zulu, Charles Michelo

**Affiliations:** 1 Department of Health Policy and Management, School of Public Health, University of Zambia, Lusaka, Zambia; 2 Department of Epidemiology & Biostatistics, School of Public Health, University of Zambia, Strategic Centre for Health Systems Metrics & Evaluations (SCHEME), Lusaka, Zambia; 3 Department of Epidemiology and Biostatistics, School of Public Health, University of Zambia, Lusaka, Zambia; 4 Department of Biostatistics, School of Public Health, University of Ghana, Accra, Ghana

**Keywords:** Fertility, Adolescent fertility, Adolescents, Socioeconomic status, Zambia

## Abstract

**Background:**

Adolescents face significant barriers to access and utilization of sexual and reproductive health services in many low-income settings, which in turn may be associated with adverse consequences such as early pregnancy, sexually transmitted infections, unsafe abortion and mortality. There is evidence suggesting that limited access to sexual and reproductive health information and services among adolescents contributes to these outcomes. We aimed to find out the factors that affect the fertility of adolescents aged 15 to 19 years in Zambia and to identify possible drivers of adolescents’ fertility.

**Methods:**

Secondary analysis of the ZDHS 2013/14 data was carried out to find out the factors that affect the fertility rate of adolescents aged 15 to 19 years using multivariate logistic regression (n = 3666).

**Results:**

Overall, 23.1% of adolescents had given birth at least once in the 5 years leading to the survey (n = 3666, 99.4% response), and 49.8% were rural-based while 50.2% were urban-based. The median number of schooling was 8 years (IQR 6–10). About 52% of the adolescents were in the poorer, poor and medium wealth quintiles while the other 48% were in the rich and richer quintiles. Factors found to affect fertility include residence, wealth status, educational attainment, marriage and abortion. An urban-based adolescent with a lower socioeconomic status was 2.4 times more likely to give birth compared to rural-based poorer adolescents (aOR = 2.4, 95% CI: 1.5, 3.7, p < 0.001). Although odds of giving birth were much higher among rural-based married adolescents (aOR = 8.0, 95% CI: 5.4, 11.9, p < 0.001) compared to urban married adolescents (aOR = 5.5, 95% CI: 8.3, 16.0, p < 0.001), and these relationships both statistically significant, higher educational attainment (aOR = 0.7, 95% CI: 0.6, 0.8 p < 0.001) and abortion (aOR = 0.3, 95% CI: 0.1, 0.8, p = 0.020) reduced these odds, particularly for rural-based adolescents.

**Conclusion:**

Despite response aimed at reducing adolescent fertility, low wealth status, low educational attainment and early marriage remain significant drivers of adolescent fertility in Zambia. There is a need to address sexual and reproductive health needs of urban-based adolescents with a lower socioeconomic status.

## Plain Language Summary

Adolescents go through serious challenges related to accessing and using sexual and reproductive health services in many low-income settings, and may also be related to negative consequences such as early pregnancy, sexually transmitted infections and unsafe abortion and death. Research has revealed that limited access to sexual and reproductive health information and services among young people contributes to these negative consequences. This analysis aimed to find out the factors that affect the fertility of 3666 adolescents aged 15 to 19 years in Zambia and to identify possible drivers of adolescents’ fertility, using the Zambia Demographic and Health Survey conducted in 2013/2014. A little over a quarter of the adolescents had given birth at least once in the five years leading to the survey. About half of the young people were rural-basedwhile the other half were urban-based, with an average of about 8 years in school. The rural-based adolescents had slightly lower average years in school compared to the urban-based; 7 years and 9 years respectively. Living in a rural area, residing in a home with a low wealth status and being married were all linked to higher chances of giving birth, while terminating a pregnancy and having more years of education were linked to lower chances of giving birth among the adolescents. Urban-based adolescents with lower wealth status were also linked to higher chances of giving birth compared to urban-based adolescents with higher wealth status. These results suggest that while residing in rural areas and being married increase the chances of higher fertility, the adolescents in urban areas but with lower wealth status also need interventions aimed at reducing their fertility.

## Introduction

The Guttmacher–Lancet Commission report on accelerating progress for sexual and reproductive health and rights (SRHR) for all states that “progress in SRHR requires confrontation of the barriers embedded in laws, policies, the economy, and in social norms and values—especially gender inequality—that prevent people from achieving sexual and reproductive health” [1]. Globally, high adolescent fertility has been a significant concern [2]. Over 16 million women aged 15–19 years give birth each year, especially in sub-Saharan Africa where 95% of these births take place [3]. Early childbearing is linked to a higher risk of maternal and child health complications [4]. The adverse health effects are more severe in rural areas, where fertility is higher [5], with lower access to social services such as education. While more years of education are noted to delay the onset of childbearing among adolescents, their fertility rates remain high [5, 6] because of limited access to both education and health services.

Educational attainment has globally been a factor in the definition of socioeconomic status, and it has often been argued that low socioeconomic status is linked to reduced access to quality education and health services. Other Socioeconomic empowerment factors; key in understanding fertility preferences among women include increased skills development, increased decision-making power, and more control over household resources [7]. Fertility challenges globally may thus be defined as a function of socioeconomic status, which may further be associated with early childbearing in LMICs [8].

Fertility rates must be reduced; to address the morbidity and mortality associated with early childbearing. Adolescent sexual and reproductive health-focused interventions have been undertaken [9], particularly in Zambia, where teenage pregnancy has had an impact on school dropouts of young pregnant girls [10]. Most of the pregnancies are unplanned thereby increasing the risk of unsafe abortions [11]. Typically, most of the adolescents are in this predicament due to low knowledge of contraception, poor access safe abortion services [11–14]. Approximately 90% of all the abortion-related deaths, along with 32% of maternal deaths are preventable by the use of effective contraception [15].

With an overall aim of improving the health status of Zambians, the Reproductive, Maternal, Newborn, Child and Adolescent Health and Nutrition Communication and Advocacy Strategy 2018–2021 aims “to effectively target and serve adolescents and youth with quality accessible sexual and reproductive health information and services in and out of school” [16]. Bearing in mind the link between poverty and high fertility, one would argue that young people with low socioeconomic status tend to suffer more severe consequences of limited access to services. The Zambian population is young; over 50% below 15 years old, and about 60% below 24 years old [17]. Childbearing begins early with more than 30% of all the women giving birth by the time they are 18 years old and about 29% of adolescents aged 15 to 19 are already mothers or are pregnant with their first child [17, 18]. Also, the social norms surrounding adolescent pregnancies injunctively view them as “unwanted, risky and dangerous” although early pregnancies and marriage are also viewed as a way to secure socioeconomic security [19]. Besides, over the last 20 years, contraception use has remained low among adolescent girls in Zambia [17, 18, 20].

A deeper understanding of the factors affecting adolescent fertility is required to fill up the dearth of information necessary to inform programs and the health care system that aims to meet the sexual and reproductive health needs of this population cohort. This study aimed to explore the determinants of adolescent fertility in Zamia.

## Methodology

### Study setting, aim and design

This study was undertaken in Zambia, a southern Africa country with approximately 13 million people and an annual growth rate of about 2.8. [21]. This analysis aimed to examine the determinants of fertility among adolescents aged 15 to 19 years in Zambia, using data from the 2013/2014 Zambia Demographic and Health Survey.

### ZDHS design

*Data* were collected during the 2013/2014 Zambia Demographic and Health Survey. The survey used a two-stage stratified cluster sample design, clusters selected during the first stage and households selected during the second stage. After dividing Zambia into ten provinces, stratification was done by splitting each province into urban and rural clusters, creating 20 sampling strata. The sampling frame consisted of 25, 631 convenient geographical areas with an average size of 130 households or 600 people. In the first stage, 722 clusters (305 in urban areas and 417 in rural areas) were selected with probability proportional to size. In the second stage, a complete list of households served as the sampling frame in the selection of households for enumeration. About 25 households were selected in each cluster. During the second stage of selection, a representative sample of 18,052 households was selected.

### Adolescent fertility design

Data on the fertility of female adolescents between 15 and 19 years old were extracted for this sub-study from the 2013/2014 Zambia Demographic and Health Survey. This selection included all female adolescents who were permanent residents of the households or visitors in the households on the night before the survey began. The total number of adolescents 15 to 19 years old included in this analysis was 3, 666, representing 22.5% of the total sample of women 15 to 49.

The variable ‘births’ was taken as the dependent variable in this analysis, defined as whether an adolescent had ever given birth or not. This study focused on ‘births’ as a binary variable (0 for no birth and 1 for at least one birth). For the explanatory variables, Bongaarts’ proximate determinants of fertility [22], as they relate to adolescents aged 15 to 19 were used to guide the extraction of data, depending on availability of the information in the data set. The main variables extracted and recoded were marriage [as a binary variable (0 for unmarried and 1 for married), marriage as a categorical variable (1 for never in union, 2 for married, 3 for living with partner, 4 for widowed, 5 for divorced and 6 for separated), contraceptive use as a binary variable (0 for no use and 1 for use), sexual activity as a binary variable (0 for never had sex, 1 for ever had sex) and abortion as a binary variable (0 for never terminated pregnancy and 1 for ever terminated a pregnancy]. This variable included those who did something to induce an abortion and did not include spontaneous abortions.

Other variables extracted were abstinence as a binary variable (0 for no abstinent and 1 for abstinent girls). In the 2013/14 ZDHS report, abstinence is defined as girls who are not sexually active and have not given birth before. Age was continuous, but was also taken as categorical since the range was narrow, age at first sex, which was considered as count data but later regrouped into the following age ranges of consent for sex; 10–14, 15, 16–19, type of residence (0 for urban and 1 for rural), educational attainment was taken as education level (0 for no education, 1 for primary, 2 for secondary education and 3 for tertiary education) and number of years in school which was regrouped to realign it into the current Zambian education system; no education, lower primary, upper primary, junior secondary, senior secondary and tertiary education). Knowledge of contraception was considered as a binary variable (0 for no knowledge and 1 for knowledge of at least one method), wealth status as a binary variable (0 for poor, 1 for rich) and wealth quintiles were considered as categorical (1 for poorer, 2 for poor, 3 medium 4 for rich and 5 for richest) and finally religion was defined as a categorical variable (1 for catholic, 2 for christian non-catholic, 3 for muslim and 4 for other). The total number of covariates was 15.

### Analysis

All analyses were conducted using Stata version 13 [23]. Data were cleaned and adjusted for multistage cluster sampling using the ‘svyset’ survey command [24, 25] in Stata since the ZDHS study adopted a complex survey design. Complex survey designs produce standard errors that are different from those obtained from the simple random sampling procedure. Standard errors are essential for confidence intervals and hypothesis testing. The data are meant to be representative of the population and are weighted. For this data set, individual weights took into account the disproportionate sampling and non-response during data collection.

During the analysis, the weights were taken into account at each stage of the analysis, ensuring nationally representative estimates. Proportions were used to describe the data. To test the association of each of the independent variables and births, univariate standard logistic regression was used to obtain the odds ratios and 95% confidence intervals (CIs). Variables that were correlated, with a correlation coefficient > 0.8 were dropped from the multivariable logistic regression. Variables that predicted failure or success perfectly were not included in the analysis. The final model was arrived at using an investigator-led backward step-wise approach and was tested using the Hosmer–Lemeshow goodness of fit test. However, variables that were relevant according to literature were maintained in the final model.

The analysis also controlled for the potential confounders: age, wealth and educational status. Age was adjusted for as a linear effect. Survey data is prone to biases such as missingness and failure to recall events that have already passed. However, incomplete and missing data were found to be low (less than 5%) enough to be inconsequential [26] and hence a complete case analysis [27] was implemented. The variables were also limited to the data sets from the survey and therefore new variables could not be added to enrich the analysis.

### Ethical considerations

In the ZDHS, All the respondents in the survey provided verbal consent when the data were collected. Authorization to survey in 2013 was granted by the Ministry of Health of Zambia and the Institutional Review Board of ICF International, and the Centers for Disease Control and Prevention (CDC) in Atlanta, USA [17]. For this study, ethical approval was sought from the Excellence in Research Ethics (ERES) IRB (REF. 2017-Apr-007) in Lusaka, Zambia in 2017.

## Results

### Participants’ characteristics

Of the total sample size (n = 3666) of the adolescents, and given that 99.4% response was recorded among all women 15 to 49 years old, non-participation was assumed to be in this range because there were almost no losses of significant records based on completeness. Details of participation rates for the 2013/14 ZDHS are published in the report [17]. Distribution was as follows: comparable rural versus urban distribution (49.8% vs. 50.2% respectively; p < 0.001); 56% had attained at least junior secondary level education, with only 1.9% of the adolescents who had never been to school.

The overall median number of schooling was 8 years (IQR 6–10) and significant rural vs. urban differences were observed (7 years [IQR 6–8] vs. 9 years [IQR 7–10] respectively (p < 0.001). Regarding wealth status, 52% of the adolescents were in the poorer, poor and medium wealth quintiles while the other 48% were in the rich and richer quintiles. About 70% of the adolescents who had sex after their first marriage had already given birth at least once.

### Fertility determinants

The determinants of adolescent fertility included marriage, knowledge of contraception, contraception use and abortion. About 23.1% of the adolescents had given birth in the last 5 years, and the number of births increased with increase in age. A majority of the adolescents (over 90%) were reported to know of contraception by mentioning at least one contraception method, while contraception use was reported at a low 10%. Over 80% of adolescents were unmarried, while 51% had had sex before. Sixty cases of adolescent girls had terminated a pregnancy (abortion), representing 1.6% of the girls included in this analysis. Abstinence was also low (9.8%) among the adolescents, despite almost half of them reporting that they had never had sex, and 30% reporting that they had not been sexually active. Further investigation revealed that abstinence was captured as post-partum abstinence. Table 1 shows the descriptive statistics of the study population.

**Table 1:** Characteristics of adolescents aged 15 to 19 in Zambia

	No	% out of n*	% at least 1 birth^a^
Socio-demographics			
Age			
15	731	19.9	3.1
16	756	20.6	7.7
17	667	18.1	17.1
18	771	21.0	35.7
19	741	20.2	51.1
Age at first sex			
10 to 14	425	11.6	42.2
15	458	12.5	45.3
16 to 19	777	21.2	40.4
Not had	1797	49	00.0
After marriage	209	5.7	70.9
Type of residence			
Rural	1827	49.8	28.7
Urban	1839	50.2	17.7
Educational attainment			
None	68	1.9	46.4
Primary	1414	38.6	28.7
Secondary	2170	59.2	18.9
Tertiary	14	0.4	14.2
Educational attainment^b^			
No education	75	2.1	45.3
Lower primary	259	7.1	33.6
Upper primary	1294	35.3	26.7
Junior secondary	1347	36.7	22.7
Senior secondary	682	18.6	10.9
Tertiary	9	0.3	11.1
Wealth quintiles			
1st	538	14.7	37.4
2nd	598	16.3	30.3
3rd	770	21	27.5
4th	825	22.5	21.6
5th	935	25.5	8.3
Wealth (rich vs poor)			
Rich (4th & 5th)	1,760	48.0	14.5
Poor (1st,2nd & 3rd)	1,906	52.0	31.1
Underlying determinants			
Religion			
Catholic	692	18.9	18.6
Protestant	2,954	80.6	24.2
Muslim	9	0.3	0.0
Other	11	0.3	36.5
Knowledge of contraception			
Does not know	162	4.4	3.7
Knows	3504	95.6	24.1
Abstinence			
No	3308	90.2	14.8
Yes	358	9.8	1
Proximate determinants			
Contraception use			
None	3278	89.4	16.4
At least a method	388	10.6	80.0
Marital status			
Single	3098	84.5	13.8
Married	568	15.5	73.9
Marital status			
Never in union	3,042	83.0	12.8
Married	553	15.1	73.8
Living with Partner	15	0.4	80.0
Widowed	5	0.1	40.0
Divorced	30	0.8	80.0
Separated	21	0.5	71.4
Sexual activity			
Never had sex	1797	49.0	0.0
Ever had sex	1869	51.0	52.2
Abortion			
No	3606	98.4	22.9
Yes	60	1.6	41.7

*n = 3,666

Births (ever given birth in the last 5 years) 849 (23.1%)

^a^The proportion of adolescents in that group with at least one birth in the last 5 years

^b^Adjusted number of years in school. Median years in school—8 years (IQR 6–9), Rural 7 years (IQR 6–8) Urban 9 years (IQR 7–10)

In the univariate variable selection procedure, age, age at first sex, residence, educational attainment, wealth status, marital status, abortion (termination of pregnancy), contraceptive use and knowledge of contraception all had a statistically significant association with adolescent births outcome. Table 2 shows the adjusted odds ratios and 95% CIs in the final model.

**Table 2:** Determinants for ‘ever giving birth’ among adolescents 15 to 19

	% out of n* (number)	Adjusted OR (95% CI)	p-value
Age			
15	19.9 (731)	1	
16	20.6 (756)	2.3 (1.3, 4.2)	0.007
17	18.1 (667)	6.4 (3.6, 11.5)	< 0.001
18	21.0 (771)	15.6 (8.9, 27.1)	< 0.001
19	20.2 (741)	29.1 (16.9, 50.1)	< 0.001
Per year increase		2.3 (2.1, 2.5)	< 0.001
Wealth (Rich vs Poor)			
Rich (4th &5th)	48.0 (1,760)	1	
Poor (1st,2nd & 3rd)	52.0 (1,906)	1.7 (1.3, 2.4)	< 0.001
Educational attainment			
No education	2.1 (75)	1	
Lower primary	7.1 (259)	0.5 (0.2, 1.2)	0.123
Upper primary	35.3 (1,294)	0.6 (0.3, 1.3)	0.228
Junior secondary	36.7 (1,347)	0.4 (0.2, 0.7)	0.006
Senior secondary	18.6 (682)	0.1 (0.0, 0.2)	< 0.001
Tertiary	0.3 (9)	0.1 (0.0, 0.8)	0.035
Knowledge of contraception			
Does not know	4.4 (162)	1	
Knows	95.6 (3,504)	5.4 (1.9, 15.6)	0.002
Marital status			
Single	84.5 (3,098)	1	< 0.001
Married	15.5 (568)	6.7 (4.9, 9.2)	
Contraception use			
None	89.4 (3,278)	1	
At least one method	10.6 (388)	14.4 (9.2, 22.4)	< 0.001
Abortion			
No	98.4 (3,606)	1	
Yes	1.6 (60)	0.5 (0.2, 1.3)	0.174

*n = 3666

Having attained junior secondary education was associated with reduced odds of having given birth, compared to those who had no education (aOR = 0.4, 95% CI: 0.2, 0.7, p = 0.006). Following adjustment for age and educational attainment, the poor adolescents were 1.7 times more likely to have given birth, compared to the rich adolescents (aOR = 1.7, 95% CI: 1.3, 2.4, p < 0.001). The odds of having given birth increased with age (aOR = 2.3, 95% CI: 2.1, 2.5, p < 0.001).

Adjusting for age, wealth and educational attainment, the odds of having given birth for adolescents were significantly higher among the adolescents who knew at least one contraceptive method, compared to those that did not know any method (aOR = 5.4, 95% CI: 1.9, 15.6, p = 0.002). Married adolescents were more likely to have given birth, compared to those who were not married, adjusting for socio-demographic variables (aOR = 6.7, 95% CI: 4.9, 9.2, p < 0.001) and this relationship was statistically significant. There was also a strong relationship between contraceptive use and giving birth (aOR = 6.7, 95% CI: 9.2, 22.4, p < 0.001) compared to those who did not use any contraceptive method, adjusting for socio-demographic variables. Abortion (ever terminated a pregnancy) was the only proximate determinant associated with reduced odds of giving birth (aOR = 0.4, 95% CI: 0.2, 1.3, p = 0.174), adjusting for socio-demographic variables. However, this relationship was not statistically significant.

We also present adolescent fertility determinants by rural and urban residence. Table 3 presents findings from this analysis. The odds of giving birth increased with age for both rural and urban adolescents; though these odds were higher for rural adolescents (aOR = 2.5, 95% CI: 2.2, 2.9, p < 0.001), in comparison to urban adolescents (aOR = 2.1, 95% CI: 1.7, 2.4, p < 0.001). Also, increase in educational attainment was associated with reduced odds of giving birth, and these odds were lower for urban adolescents (aOR = 0.7, 95% CI: 0.6, 0.8, p < 0.001) compared to rural adolescents (aOR = 0.5, 95% CI: 0.3, 0.6, p < 0.001). Interestingly, following adjustment for age and educational attainment, poor urban adolescents had higher odds of giving birth, compared to the rich urban adolescents (aOR = 2.4, 95% CI: 1.5, 3.7, p < 0.001).

**Table 3:** Determinants for ‘ever giving birth’ among adolescents stratified by residence

Characteristic description	No	% out of n	Rural		Urban	
			Adjusted OR	p-value	Adjusted OR	p-value
			(95% CI)		(95% CI)	
Age						
15	731	19.9				
16	756	20.6				
17	667	18.1	2.5 (2.2, 2.9)^a^	< 0.001	2.1 (1.8, 2.4)	
18	771	21				
19	741	20.2				
Wealth (rich vs poor)						
Rich (4th and 5th)	1,760	48	1		1	
Poor (1st, 2nd and 3rd)	1,906	52	1.3 (0.8, 2.0)	0.23	2.4 (1.5, 3.7)	
Educational attainment						
No education	75	2.1				
Lower primary	259	7.1				
Upper primary	1,294	35.3	0.7 (0.6, 0.8)		0.5 (0.3, 0.6)	
Junior secondary	1,347	36.7				
Senior secondary	682	18.6				
Tertiary	9	0.3				
Knowledge of contraception						
Does not know	162	4.4	1			
Knows	3,504	95.6	4.6 (1.4, 15.2)	0.014		*
Marital status						
Single	3,098	84.5	1		1	
Married	568	15.5	8.0 (5.4, 11.9)	< 0.001	5.5 (3.2, 9.4)	< 0.001
Contraception use						
None	3,278	89.4	1		1	
At least one method	388	10.6	13.8 (7.4 19.9)	< 0.001	15.8 (8.3, 16.0)	< 0.001
Abortion						
No	3,606	98.4	1		1	
Yes	60	1.6	0.3 (0.1, 0.8)	0.02	0.6 (0.2, 9.2)	0.717

n = 3666

^a^Per year increase

*There were no urban adolescents without knowledge of contraception

Adjusting for age, wealth and educational attainment, the odds of giving birth for rural adolescents were significantly higher among the adolescents who knew at least one contraceptive method, compared to those that did not know any method (aOR = 4.6, 95% CI: 1.4, 15.2, p = 0.014). Interestingly, there were no urban adolescents who and given birth and did not know at least one contraceptive method. While married adolescents were more likely to give birth, compared to the unmarried, adjusting for socio-demographic variables, the odds of giving birth were much higher among rural married adolescents (aOR = 8.0, 95% CI: 8.0, 5.4, 11.9, p < 0.001) compared to urban married adolescents (aOR = 5.5, 95% CI: 8.3, 16.0, p < 0.001), and these relationship were both statistically significant.

In addition, and adjusting for socio-demographic variables, the odds of giving birth and using at least one contraceptive method were significantly higher among urban adolescents (aOR = 15.8, 95% CI: 8.3, 16.0, p < 0.001) compared to rural adolescents (aOR = 13.8, 95% CI: 7.4, 19.9, p < 0.001). Finally, abortion (ever terminated a pregnancy) among rural adolescents was significantly associated with reduced odds of giving birth (aOR = 0.3, 95% CI: 0.1, 0.8, p < 0.020), adjusting for socio-demographic variables.

## Discussion

We report a high proportion of adolescents who had ever given birth (in the last 5 years leading to the survey) than expected and a further look at the observation shows that being in a lower-income household alone was associated with increased chances of giving birth, while higher educational attainment reduced these chances when other factors were adjusted. These findings also consistently show these patterns of association for both rural and urban areas, suggesting a strong link between socio-economic status and fertility in general, and further indicating the presence of differential inequities that may be at play in the study population. Other studies have indicated similar observations among adolescent boys and girls [5, 8, 28–30].

Higher wealth status increased protection against ever giving birth among the adolescents, particularly for those based in urban areas. A study in rural Zambia highlighted that early childbearing was often a source of economic and social security, while schooling was viewed as a deterrent of early child bearing [19]. Remarkably, we observed that urban adolescents with a lower wealth status were more likely to give birth compared to rural adolescents. Others found that advantageous socioeconomic status increased the likelihood of postponement of childbearing among adolescents and young adults [31]. The link between low wealth status and ever giving birth could be an indication of reduced accessibility to contraception information and services among adolescents with a lower wealth status. A study observed that despite the high potential for coverage of social services and family planning, in this case, the urban adolescents with a lower wealth status still have inaccessible or low-quality services and a low response to the marginalizing effects of their socioeconomic status [32].

Higher educational attainment was strongly linked to reduced odds of ever giving birth. In addition to reduced fertility, literature also suggests that higher educational attainment is linked to higher wealth status [33], suggesting that these are essential drivers of adolescent fertility that need to be looked into further. Keeping in mind the empowerment factors that are important for understanding fertility preferences among women- higher education, increased skills development, increased decision-making power, and more control over household resources [7], a case for keeping girls in school to reduce their fertility can be made, with a specific focus on rural and urban-poor adolescents who face reduced empowerment and severe income inequalities [34].

Marital status was another significant determinant of adolescent fertility in this study; married adolescents were more likely to give birth compared to the unmarried. There is an indication that marriage continues to contribute to early childbearing, especially with 70% of the married adolescents already giving birth at least once. These findings were consistent with evidence from 24 African countries [35, 36]. Marriage increases childbearing expectations of the adolescents by their communities as is the case in many African cultures [37] and marriage is viewed as a form of security for the young daughters from rape, premarital sexual activity, unintended pregnancy outside marriage and infections [38]. Furthermore, rural married adolescents were more likely to give birth compared to urban married adolescents and this was consistent with studies done elsewhere [35].

While marriage was linked to increased odds of ever giving birth, these odds were also linked to contraception use, indicating that ever giving birth increased access to SRH services for adolescents and the health systems responds more married adolescents and those who had ever given birth compared to those that had not. Also, studies have found that married adolescents are less likely to face stigma for early pregnancy, accessing contraceptives and sexual and reproductive health services due to their marital status [37]. This observation could explain why contraception use was much higher among the adolescents who had given birth before or were married, compared to adolescents who were not. Besides, family planning interventions in Zambia have gradually been shifting towards providing postpartum contraception information and services to women and girls as they go for antenatal services to increase child spacing and reduce fertility [39]. Despite this intervention, this finding indicates that unmarried adolescents, who had never given birth before remained unreached and at risk of unwanted pregnancy and unsafe abortions [11, 40, 41] and their situation is compounded by lower socioeconomic status and lower educational attainment.

The ZDHS reports indicate that knowledge of contraception is ‘universal', and our observation too was that there was a significant relationship between knowledge of contraception and ever giving birth, especially in rural areas. Paradoxically, it has been noted in other studies that adolescents have inadequate information about conception, contraception and abortion [14, 42, 43] and they remain making fertility control-related decisions based on incomplete or inadequate information about contraception [11, 40, 44]. Knowledge among rural adolescents was significantly linked to reduced odds of ever giving birth while all the urban adolescents knew at least one contraceptive method. Rural/ urban differences in knowledge indicate the need for different approaches for these adolescents and their unique SRH needs.

Zambian health system’s response through postpartum contraception [39] has been effective to an extent, but also too late for those who have not given birth before as they continue to face barriers to information and services while adolescents who have given birth before are in a better position to learn about contraception, although postpartum. Alternatively, the link between increased odds of giving birth and contraceptive knowledge and use could also be an indication of other socio-cultural factors that could affect the uptake of contraception among young people who have never given birth before such as the fear of infertility and fear of side effects of contraception, or alternative sources of contraception such as traditional healers [45]. The 2013/2014 ZDHS reports that adolescents 15 to 19 had the highest unmet need for contraception (25%) compared to those in older age groups, which marginally reduced to 21% in 2018 [17, 18]. Nonetheless, there is a need to probe further on both the knowledge and use of contraception among adolescents to reduce the barriers to contraception access and utilization. A systematic review of contraception knowledge, attitudes and practices of adolescents in low and middle income countries revealed that young people mostly used condoms due to their accessibility, and this was higher among boys compared to girls [45].

Interestingly, abortion had a significant relationship with reduced fertility for rural-based adolescents although they were expected to have had limited access to safe abortion services, and this could be an indicator of unsafe abortion among rural adolescents. A study in rural India reported that abortions were the preferred method for fertility control [46]. The findings from this study could be indicative of abortion as an option for those who had given birth before, even though only 1.6% of the adolescents in the survey had ever terminated a pregnancy. However, abortion information is usually underreported [47–49]. Literature suggests that abortion rates are higher than this for adolescents but the incidence in Zambia is still unknown, and it is mostly estimated from facility-based post-abortion care information, or from selected facilities that have support for the provision of abortion care services [50].

While some interventions are on occasion inclusive of the rural-based adolescents from lower-income households, urban-based adolescents from lower-income households also need specific interventions that respond to their unique structural position. Additionally, an increase in age was significantly associated with giving birth, particularly for rural-based adolescents and this was consistent with a study in Ethiopia [36]. This study suggests that rural older adolescents are more likely to experience childbearing, compared to the younger ones. Differences in the chance of giving birth by age indicate a need to age-appropriate messages and interventions for the varying age-related SRH needs rather than grouping them into one homogeneous group [35]. Others stressed the need to focus on different categories of adolescents in the various interventions as they have varying sexual and reproductive health information and needs [51]. Also, addressing urban poverty and access to services in these communities then becomes a significant factor in controlling fertility [1, 52]. In India, school-based life skills training programs have been noted as effective in increasing agency and the socioeconomic status of girls, while encouraging them to stay in school [53].

### Limitations

In the 2013/2014 ZDHS, the ‘births’ variable has three levels, reporting those who had given birth up to at least 3 times. In this analysis, the variable 'births' was binary to ensure an adequate number of observations for the analysis. This analysis was based on a cross-sectional survey that included questions that were answered retrospectively. As such, causality could not be established, and there was uncertainty on the exact estimates as respondents were asked to recall past events. Besides, some of the data may have been under-reported due to the discomfort of discussing sensitive topics such as abortion among adolescents. This may have led to an underestimation of some variables such as abortion. However, abortion could be evaluated further to see its relationship with adolescent fertility. Despite these limitations, the study results remain valid and are useful for informing policy and practice, as well as a basis for more research on adolescent fertility. The results from this analysis are based on a sample with national representation; therefore, are generalizable to all locations in Zambia.

## Conclusion

In conclusion, finding that births were higher in lower socioeconomic groups while groups with higher educational attainment were significantly linked to lower chances of giving birth among adolescents suggests a key indicator of the linkage between lower poverty levels and reduced fertility. Also, this suggests that the bulk of past and current programmatic response to the high fertility rates in Zambia may have taken a supermarket approach, missing those most in need and at risk- lowly educated and predominantly poor adolescents, who are also more likely to be married- another significant driver of fertility. The fact that most of these programs may also be concentrated in urban areas may further suggest the presence of systemic supply-driven programmatic inequality thus widening the gap even more. The morality of this is what must be questioned, examined further and curtailed. The inherent problematic nature of the response is evident in that the provision of contraception to young people who have given birth is present, but the adolescents only know about and use contraception when they have already given birth at least once, indicating a delayed response to the fertility control needs of the adolescent.

These findings suggest a critical need of policy shift and adjustment to target most-at-risk adolescents, such as urban-based adolescents with a lower socioeconomic status, rural adolescents, as well as repacking contraception awareness programs in mediums that are acceptable and understood by most of the population. Specific policy focus could draw lessons from interventions that have been successful elsewhere-; multi-sectoral strategies for keeping girls in school, rolling out Comprehensive Sexuality Education and an increase in the provision of inclusive youth-friendly SRH services that delay the onset of childbearing adolescents and delays subsequent pregnancies for those who have given birth before. In general, this calls for context-specific programming, informed by context differentials but grounded in messaging and communication that resonates well with the adolescents and the rest of the community members. This innovation is what will help reach out to adolescents in the different socio-cultural contexts also including other population subgroups, thereby providing a specific response to the different and unique groups of adolescents in Zambia- and attaining the Universal Health Coverage goals of leaving no one behind.

## Data Availability

The datasets used and/or analyzed for the current study are available on request on the Demographic and Health Surveys (DHS) Program website. The link is https://dhsprogram.com/

## Abbreviations

aOR: Adjusted odds ratio
IQR: Inter quartile range
LMIC: Low and lower middle income countries
SRH: Sexual and reproductive health
ZDHS: Zamia Demographic and Health Survey
